# Supplementary material for: Cardiometabolic Traits in Adult Twins: Heritability and BMI Impact with Age
Source: Nutrients. 2022 Dec 29;15(1):164. doi: 10.3390/nu15010164 (PMC9824881; doi:10.3390/nu15010164)
Supplement: Supplementary file 1 [file nutrients-15-00164-s001.zip › nutrients-2082499-supplementary.pdf]

Supplementary Table S1 Assumptions testing of univariate SEM for BMI and cardiometabolic risk factors in full analysis population

Model	ep	-2LL	df	AIC	Δdf	P-values
**BMI**						
Saturated model	12	5769.18	2292	5793.18		
Submodel 1	10	5769.76	2294	5789.76	2	0.75
Submodel 2	9	5769.86	2295	5787.86	3	0.88
Submodel 3	7	5770.81	2297	5784.81	5	0.90
Submodel 4	6	5778.61	2298	5790.61	6	0.15
**HbA1c**						
Saturated model	13	4780.46	1981	4806.46		
Submodel 1	11	4780.54	1983	4802.54	2	0.96
Submodel 2	10	4781.81	1984	4801.81	3	0.72
Submodel 3	8	4789.57	1986	4805.57	5	0.10
Submodel 4	7	4794.30	1987	4808.30	6	0.03
**FBG**						
Saturated model	13	5435.68	2163	5461.68		
Submodel 1	11	5437.42	2165	5459.42	2	0.42
Submodel 2	10	5438.19	2166	5458.19	3	0.48
Submodel 3	8	5439.84	2168	5455.84	5	0.53
Submodel 4	7	5452.15	2169	5466.15	6	0.01
**SBP**						
Saturated model	12	5730.01	2300	5754.01		
Submodel 1	10	5731.37	2302	5751.37	2	0.51
Submodel 2	9	5732.20	2303	5750.20	3	0.54
Submodel 3	7	5732.82	2305	5746.82	5	0.73
Submodel 4	6	5738.12	2306	5750.12	6	0.23
**DBP**						
Saturated model	12	6013.08	2330	6037.08		
Submodel 1	10	6015.30	2332	6035.30	2	0.33
Submodel 2	9	6015.74	2333	6033.74	3	0.45
Submodel 3	7	6020.49	2335	6034.49	5	0.19
Submodel 4	6	6022.53	2336	6034.53	6	0.15
**TC**						
Saturated model	13	5905.87	2247	5931.87		
Submodel 1	11	5906.36	2249	5928.36	2	0.78
Submodel 2	10	5907.40	2250	5927.40	3	0.68
Submodel 3	8	5909.50	2252	5925.50	5	0.60
Submodel 4	7	5910.43	2253	5924.43	6	0.60
**TG**						
Saturated model	13	5972.11	2213	5998.11		

Model	ep	-2LL	df	AIC	Δdf	P-values
Submodel 1	11	5972.70	2215	5994.70	2	0.74
Submodel 2	10	5973.93	2216	5993.93	3	0.61
Submodel 3	8	5975.18	2218	5991.18	5	0.69
Submodel 4	7	5975.37	2219	5989.37	6	0.78
**LDL-C**						
Saturated model	13	6040.41	2253	6066.41		
Submodel 1	11	6040.72	2255	6062.72	2	0.86
Submodel 2	10	6040.87	2256	6060.87	3	0.93
Submodel 3	8	6049.44	2258	6065.44	5	0.11
Submodel 4	7	6049.82	2259	6063.82	6	0.15
**HDL-C**						
Saturated model	13	5781.04	2251	5807.04		
Submodel 1	11	5781.20	2253	5803.20	2	0.92
Submodel 2	10	5781.51	2254	5801.51	3	0.93
Submodel 3	8	5783.00	2256	5799.00	5	0.86
Submodel 4	7	5783.08	2257	5797.08	6	0.92

Abbreviations: BMI, body mass index; HbA1c, glycated hemoglobin; FBG, fasting blood glucose; SBP, systolic blood pressure; DBP, diastolic blood pressure; TC, total cholesterol; TG, plasma triglyceride; LDL-C, low-density lipoprotein-cholesterol; HDL-C, high-density lipoprotein-cholesterol.

*ep, parameters that were free to be estimated; -2LL, negative log-likelihood; df, degrees of freedom; AIC, Akaike's Information Criteria, an alternative fit index. Lower values indicate better model fits; Δdf, the difference in degrees of freedom compared to the saturated model.

*Saturated model, baseline models that estimate the variances and means separately for each twin in a pair and across zygosity; Submodel 1 equates means within twin pairs; Submodel 2 equates means across zygosity; Submodel 3 equates variances within twin pairs; Submodel 4 equates variances across zygosity.

Supplementary Table S2 Twin correlations and 95%CI by zygosity from univariate SEM

	MZ	DZ
BMI	0.70(0.67,0.74)	0.39(0.30,0.48)
HbA1c	0.52(0.46,0.57)	0.32(0.22,0.42)
FBG	0.51(0.46,0.56)	0.23(0.12,0.33)
SBP	0.57(0.52,0.61)	0.33(0.23,0.42)
DBP	0.59(0.54,0.63)	0.36(0.27,0.45)
TC	0.63(0.58,0.66)	0.37(0.27,0.46)
TG	0.58(0.53,0.62)	0.33(0.23,0.42)
LDL-C	0.60(0.55,0.64)	0.32(0.22,0.41)
HDL-C	0.69(0.65,0.72)	0.41(0.32,0.49)

Abbreviations: MZ, monozygotic twins; DZ, dizygotic twins.

Supplementary Table S3 Univariate SEM fits statistics for BMI and cardiometabolic risk factors in full analysis population

Model	Comparison Model	ep	-2LL	df	AIC	Δdf	P-values
**BMI**							
Saturated model		12	5769.18	2292	5793.18		
ACE	Saturated model	7	5778.51	2297	5792.51	5	0.10
AE	ACE	6	5778.51	2298	5790.51	1	1.00
CE	ACE	6	5882.13	2298	5894.13	1	<0.01
E	ACE	5	6394.94	2299	6404.94	2	<0.01
Hom	AE	4	5790.83	2230	5798.83	2	<0.01
**HbA1c**							
Saturated model		13	4780.46	1981	4806.46		
ACE	Saturated model	8	4793.18	1986	4809.18	5	0.03
AE	ACE	7	4796.18	1987	4810.18	1	0.08
CE	ACE	7	4801.16	1987	4815.16	1	0.00
E	ACE	6	5031.75	1988	5043.75	2	0.00
Hom	ACE	5	4886.21	1989	4896.21	3	<0.01
**FBG**							
Saturated model		13	5435.68	2163	5461.68		
ADE	Saturated model	8	5451.38	2168	5467.38	5	0.01
AE	ADE	7	5451.60	2169	5465.60	1	0.64
DE	ADE	7	5466.19	2169	5480.19	1	0.00
E	ADE	6	5687.84	2170	5699.84	2	0.00
Hom	AE	5	5503.54	2171	5513.54	3	<0.01
**SBP**							
Saturated model		12	5730.01	2300	5754.01		
ACE	Saturated model	7	5737.41	2305	5751.41	5	0.19
AE	ACE	6	5739.88	2306	5751.88	1	0.12
CE	ACE	6	5754.85	2306	5766.85	1	<0.01
E	ACE	5	6097.46	2307	6107.46	2	<0.01
Hom	ACE	4	5783.07	2308	5791.07	3	<0.01
**DBP**							
Saturated model		12	6013.08	2330	6037.08		
ACE	Saturated model	7	6022.04	2335	6036.04	5	0.11
AE	ACE	6	6025.64	2336	6037.64	1	0.06
CE	ACE	6	6041.59	2336	6053.59	1	<0.01
E	ACE	5	6418.32	2337	6428.32	2	<0.01

Model	Comparison Model	ep	-2LL	df	AIC	Δdf	P-values
Hom	ACE	4	6033.50	2338	6041.50	3	0.01
**TC**							
Saturated model		13	5905.87	2247	5931.87		
ACE	Saturated model	8	5909.50	2252	5925.50	5	0.60
AE	ACE	7	5910.80	2253	5924.80	1	0.25
CE	ACE	7	5945.69	2253	5959.69	1	0.00
E	ACE	6	6363.97	2254	6375.97	2	0.00
Hom	AE	5	5911.68	2255	5921.68	2	0.65
**TG**							
Saturated model		13	5972.11	2213	5998.11		
ACE	Saturated model	8	5974.07	2218	5990.07	5	0.85
AE	ACE	7	5974.75	2219	5988.75	1	0.41
CE	ACE	7	6003.87	2219	6017.87	1	0.00
E	ACE	6	6327.49	2220	6339.49	2	0.00
Hom	AE	5	5974.77	2221	5984.77	2	0.99
**LDL-C**							
Saturated model		13	6040.41	2253	6066.41		
ACE	Saturated model	8	6049.68	2258	6065.68	5	0.10
AE	ACE	7	6049.90	2259	6063.90	1	0.64
CE	ACE	7	6086.31	2259	6100.31	1	0.00
E	ACE	6	6438.39	2260	6450.39	2	0.00
Hom	AE	5	6049.95	2261	6059.95	2	0.97
**HDL-C**							
Saturated model		13	5781.04	2251	5807.04		
ACE	Saturated model	8	5782.80	2256	5798.80	5	0.88
AE	ACE	7	5784.78	2257	5798.78	1	0.16
CE	ACE	7	5836.44	2257	5850.44	1	0.00
E	ACE	6	6351.75	2258	6363.75	2	0.00
Hom	AE	5	5786.54	2259	5796.54	2	0.41

Abbreviations: BMI, body mass index; HbA1c, glycated hemoglobin; FBG, fasting blood glucose; SBP, systolic blood pressure; DBP, diastolic blood pressure; TC, total cholesterol; TG, plasma triglyceride; LDL-C, low-density lipoprotein-cholesterol; HDL-C, high-density lipoprotein-cholesterol.

*ep, parameters that were free to be estimated; -2LL, negative log-likelihood; df, degrees of freedom; AIC, Akaike's Information Criteria, an alternative fit index. Lower values indicate better model fits; Δdf, the difference in degrees of freedom compared to the saturated model.

*Saturated model, baseline models that estimate the variances and means separately for each twin in a pair and across zygosity; A, additive genetic variance; D, nonadditive genetic variance; C, common environmental variance; E, unique environmental variance; Hom, homogeneity model, which equates the variance components across age groups.

Supplementary Table S4 Heritability estimates and 95% CI in best-fitting univariate models for BMI and cardiometabolic traits by sex

	Male	Female	$P_{homogeneity}$
BMI	0.71(0.67,0.75)	0.73(0.67,0.78)	0.77
HbA1c	0.53(0.46,0.59)	0.45(0.35,0.54)	0.08
FBG	0.50(0.44,0.56)	0.46(0.36,0.55)	0.15
SBP	0.54(0.48,0.59)	0.59(0.51,0.66)	0.12
DBP	0.58(0.52,0.62)	0.58(0.50,0.65)	0.88
TC	0.64(0.59,0.68)	0.58(0.50,0.65)	0.23
TG	0.54(0.48,0.60)	0.62(0.54,0.68)	0.14
LDL-C	0.57(0.51,0.62)	0.65(0.58,0.71)	0.05
HDL-C	0.68(0.63,0.72)	0.70(0.64,0.75)	0.39

Supplementary Table S5 Assumptions testing of univariate SEM for BMI and cardiometabolic risk factors by age group

Model	ep	-2LL	df	AIC	Δdf	P-values
**BMI**						
18-50						
Saturated model	12	3354.66	1338	3378.66		
Submodel 1	10	3356.36	1340	3376.36	2	0.43
Submodel 2	9	3356.38	1341	3374.38	3	0.63
Submodel 3	7	3357.54	1343	3371.54	5	0.72
Submodel 4	6	3361.66	1344	3373.66	6	0.32
50-60						
Saturated model	12	1473.93	572	1497.93		
Submodel 1	10	1476.91	574	1496.91	2	0.23
Submodel 2	9	1477.61	575	1495.61	3	0.30
Submodel 3	7	1477.75	577	1491.75	5	0.58
Submodel 4	6	1481.60	578	1493.60	6	0.26
>60						
Saturated model	12	929.73	358	953.73		
Submodel 1	10	929.91	360	949.91	2	0.92
Submodel 2	9	933.80	361	951.80	3	0.25
Submodel 3	7	935.02	363	949.02	5	0.38
Submodel 4	6	935.04	364	947.04	6	0.50
**HbA1c**						
18-50						

Model	ep	-2LL	df	AIC	Δdf	P-values
Saturated model	13	2692.73	1143	2718.73		
Submodel 1	11	2697.59	1145	2719.59	2	0.09
Submodel 2	10	2699.51	1146	2719.51	3	0.08
Submodel 3	8	2706.07	1148	2722.07	5	0.02
Submodel 4	7	2708.40	1149	2722.40	6	0.02
50-60						
Saturated model	13	1230.87	465	1256.87		
Submodel 1	11	1231.50	467	1253.50	2	0.73
Submodel 2	10	1231.82	468	1251.82	3	0.81
Submodel 3	8	1234.56	470	1250.56	5	0.59
Submodel 4	7	1235.79	471	1249.79	6	0.55
>60						
Saturated model	13	860.92	347	886.92		
Submodel 1	11	862.47	349	884.47	2	0.46
Submodel 2	10	862.47	350	882.47	3	0.67
Submodel 3	8	864.25	352	880.25	5	0.65
Submodel 4	7	864.51	353	878.51	6	0.73
**FBG**						
18-50						
Saturated model	13	3297.24	1285	3323.24		
Submodel 1	11	3299.71	1287	3321.71	2	0.29
Submodel 2	10	3299.98	1288	3319.98	3	0.43
Submodel 3	8	3305.64	1290	3321.64	5	0.14
Submodel 4	7	3307.53	1291	3321.53	6	0.11
50-60						
Saturated model	13	1301.62	511	1327.62		
Submodel 1	11	1303.99	513	1325.99	2	0.30
Submodel 2	10	1307.37	514	1327.37	3	0.12
Submodel 3	8	1312.65	516	1328.65	5	0.05
Submodel 4	7	1321.75	517	1335.75	6	0.00
>60						
Saturated model	13	867.25	341	893.25		
Submodel 1	11	868.17	343	890.17	2	0.63
Submodel 2	10	868.61	344	888.61	3	0.72
Submodel 3	8	872.94	346	888.94	5	0.34
Submodel 4	7	873.18	347	887.18	6	0.43
**SBP**						
18-50						
Saturated model	12	3394.79	1350	3418.79		
Submodel 1	10	3396.95	1352	3416.95	2	0.34
Submodel 2	9	3397.30	1353	3415.30	3	0.47

Model	ep	-2LL	df	AIC	Δdf	P-values
Submodel 3	7	3397.63	1355	3411.63	5	0.72
Submodel 4	6	3400.66	1356	3412.66	6	0.44
50-60						
Saturated model	12	1539.17	568	1563.17		
Submodel 1	10	1539.65	570	1559.65	2	0.78
Submodel 2	9	1539.89	571	1557.89	3	0.87
Submodel 3	7	1540.82	573	1554.82	5	0.90
Submodel 4	6	1540.83	574	1552.83	6	0.95
>60						
Saturated model	12	1010.51	358	1034.51		
Submodel 1	10	1010.61	360	1030.61	2	0.95
Submodel 2	9	1013.74	361	1031.74	3	0.36
Submodel 3	7	1014.39	363	1028.39	5	0.57
Submodel 4	6	1015.20	364	1027.20	6	0.58
**DBP**						
18-50						
Saturated model	12	3400.08	1348	3424.08		
Submodel 1	10	3401.19	1350	3421.19	2	0.57
Submodel 2	9	3401.19	1351	3419.19	3	0.77
Submodel 3	7	3404.64	1353	3418.64	5	0.47
Submodel 4	6	3408.41	1354	3420.41	6	0.21
50-60						
Saturated model	12	1583.29	584	1607.29		
Submodel 1	10	1585.51	586	1605.51	2	0.33
Submodel 2	9	1585.57	587	1603.57	3	0.51
Submodel 3	7	1588.26	589	1602.26	5	0.42
Submodel 4	6	1588.36	590	1600.36	6	0.53
>60						
Saturated model	12	1023.39	374	1047.39		
Submodel 1	10	1023.50	376	1043.50	2	0.94
Submodel 2	9	1025.62	377	1043.62	3	0.53
Submodel 3	7	1026.76	379	1040.76	5	0.64
Submodel 4	6	1026.76	380	1038.76	6	0.76
**TC**						
18-50						
Saturated model	13	3389.12	1309	3415.12		
Submodel 1	11	3389.25	1311	3411.25	2	0.94
Submodel 2	10	3389.48	1312	3409.48	3	0.95
Submodel 3	8	3390.64	1314	3406.64	5	0.91
Submodel 4	7	3390.73	1315	3404.73	6	0.95
50-60						
Saturated	13	1500.63	553	1526.63		

Model	ep	-2LL	df	AIC	Δdf	P-values
model						
Submodel 1	11	1500.73	555	1522.73	2	0.95
Submodel 2	10	1505.10	556	1525.10	3	0.21
Submodel 3	8	1506.08	558	1522.08	5	0.36
Submodel 4	7	1506.35	559	1520.35	6	0.45
>60						
Saturated model	13	972.44	359	998.44		
Submodel 1	11	972.88	361	994.88	2	0.80
Submodel 2	10	974.04	362	994.04	3	0.66
Submodel 3	8	975.72	364	991.72	5	0.66
Submodel 4	7	978.17	365	992.17	6	0.45
**TG**						
18-50						
Saturated model	13	3372.28	1281	3398.28		
Submodel 1	11	3374.21	1283	3396.21	2	0.38
Submodel 2	10	3374.22	1284	3394.22	3	0.59
Submodel 3	8	3375.70	1286	3391.70	5	0.64
Submodel 4	7	3375.72	1287	3389.72	6	0.75
50-60						
Saturated model	13	1537.57	549	1563.57		
Submodel 1	11	1541.33	551	1563.33	2	0.15
Submodel 2	10	1546.08	552	1566.08	3	0.04
Submodel 3	8	1549.58	554	1565.58	5	0.03
Submodel 4	7	1549.78	555	1563.78	6	0.06
>60						
Saturated model	13	992.06	357	1018.06		
Submodel 1	11	996.10	359	1018.10	2	0.13
Submodel 2	10	996.11	360	1016.11	3	0.26
Submodel 3	8	999.64	362	1015.64	5	0.18
Submodel 4	7	1000.04	363	1014.04	6	0.24
**LDL-C**						
18-50						
Saturated model	13	3481.90	1319	3507.90		
Submodel 1	11	3482.61	1321	3504.61	2	0.70
Submodel 2	10	3482.73	1322	3502.73	3	0.84
Submodel 3	8	3492.72	1324	3508.72	5	0.06
Submodel 4	7	3492.92	1325	3506.92	6	0.09
50-60						
Saturated model	13	1551.64	555	1577.64		
Submodel 1	11	1552.26	557	1574.26	2	0.73
Submodel 2	10	1552.26	558	1572.26	3	0.89
Submodel 3	8	1554.20	560	1570.20	5	0.77

Model	ep	-2LL	df	AIC	Δdf	P-values
Submodel 4	7	1554.35	561	1568.35	6	0.84
>60						
Saturated model	13	975.64	353	1001.64		
Submodel 1	11	977.29	355	999.29	2	0.44
Submodel 2	10	977.35	356	997.35	3	0.63
Submodel 3	8	978.66	358	994.66	5	0.70
Submodel 4	7	979.15	359	993.15	6	0.74
**HDL-C**						
18-50						
Saturated model	13	3381.07	1315	3407.07		
Submodel 1	11	3381.34	1317	3403.34	2	0.87
Submodel 2	10	3381.50	1318	3401.50	3	0.93
Submodel 3	8	3385.59	1320	3401.59	5	0.48
Submodel 4	7	3385.63	1321	3399.63	6	0.60
50-60						
Saturated model	13	1486.35	557	1512.35		
Submodel 1	11	1486.84	559	1508.84	2	0.78
Submodel 2	10	1487.31	560	1507.31	3	0.81
Submodel 3	8	1487.79	562	1503.79	5	0.92
Submodel 4	7	1487.80	563	1501.80	6	0.96
>60						
Saturated model	13	913.91	353	939.91		
Submodel 1	11	915.10	355	937.10	2	0.55
Submodel 2	10	918.21	356	938.21	3	0.23
Submodel 3	8	919.40	358	935.40	5	0.36
Submodel 4	7	919.46	359	933.46	6	0.47

Abbreviations: BMI, body mass index; HbA1c, glycated hemoglobin; FBG, fasting blood glucose; SBP, systolic blood pressure; DBP, diastolic blood pressure; TC, total cholesterol; TG, plasma triglyceride; LDL-C, low-density lipoprotein-cholesterol; HDL-C, high-density lipoprotein-cholesterol.

*ep, parameters that were free to be estimated; -2LL, negative log-likelihood; df, degrees of freedom; AIC, Akaike's Information Criteria, an alternative fit index. Lower values indicate better model fits; Δdf, the difference in degrees of freedom compared to the saturated model.

*Saturated model, baseline models that estimate the variances and means separately for each twin in a pair and across zygosity; Submodel 1 equates means within twin pairs; Submodel 2 equates means across zygosity; Submodel 3 equates variances within twin pairs; Submodel 4 equates variances across zygosity.

Supplementary Table S6 Twin correlations and 95%CI by zygosity from univariate SEM by age groups

	MZ	DZ
**BMI**		
18-50	0.72(0.67,0.76)	0.41(0.29,0.51)
50-60	0.66(0.58,0.73)	0.32(0.11,0.50)
>60	0.61(0.50,0.70)	0.23(0.00,0.48)
**HbA1c**		
18-50	0.68(0.62,0.73)	0.37(0.24,0.49)
50-60	0.33(0.18,0.46)	0.29(0.05,0.49)
>60	0.34(0.18,0.48)	0.17(0.00,0.43)
**FBG**		
18-50	0.55(0.48,0.61)	0.33(0.20,0.44)
50-60	0.48(0.36,0.59)	0.07(0.00,0.30)
>60	0.42(0.24,0.54)	0.09(0.00,0.39)
**SBP**		
18-50	0.63(0.57,0.68)	0.32(0.19,0.43)
50-60	0.56(0.47,0.65)	0.33(0.12,0.50)
>60	0.42(0.28,0.53)	0.41(0.08,0.62)
**DBP**		
18-50	0.59(0.53,0.65)	0.30(0.17,0.41)
50-60	0.56(0.46,0.64)	0.45(0.27,0.59)
>60	0.53(0.40,0.63)	0.25(0.00,0.48)
**TC**		
18-50	0.67(0.62,0.71)	0.33(0.21,0.44)
50-60	0.59(0.47,0.68)	0.28(0.00,0.53)
>60	0.59(0.47,0.68)	0.25(0.00,0.51)
**TG**		
18-50	0.61(0.55,0.66)	0.35(0.23,0.46)
50-60	0.51(0.40,0.60)	0.14(0.00,0.35)
>60	0.47(0.32,0.58)	0.24(0.00,0.47)
**LDL-C**		
18-50	0.66(0.60,0.71)	0.27(0.14,0.39)
50-60	0.46(0.35,0.56)	0.41(0.21,0.57)
>60	0.55(0.43,0.65)	0.37(0.05,0.58)
**HDL-C**		
18-50	0.71(0.66,0.75)	0.41(0.29,0.51)
50-60	0.62(0.53,0.69)	0.45(0.26,0.59)
>60	0.69(0.60,0.77)	0.27(0.00,0.51)

Abbreviations: MZ, monozygotic twins; DZ, dizygotic twins.

Supplementary Table S7 Univariate SEM fits statistics for BMI and cardiometabolic risk factors by age group

Model	Comparison Model	ep	-2LL	df	AIC	Δdf	P-values
**BMI**							
18-50							
Saturated model		12	3354.66	1338	3378.66		
ACE	Saturated model	7	3361.64	1343	3375.64	5	0.22

Model	Comparison Model	ep	-2LL	df	AIC	Δdf	P-values
AE	ACE	6	3361.67	1344	3373.67	1	0.86
CE	ACE	6	3426.57	1344	3438.57	1	<0.01
E	ACE	5	3735.45	1345	3745.45	2	<0.01
50-60							
Saturated model		12	1473.93	572	1497.93		
ACE	Saturated model	7	1480.77	577	1494.77	5	0.23
AE	ACE	6	1481.36	578	1493.36	1	0.44
CE	ACE	6	1482.13	578	1494.13	1	0.24
E	ACE	5	1612.91	579	1622.91	2	<0.01
>60							
Saturated model		12	929.73	358	953.73		
ADE	Saturated model	7	931.19	363	945.19	5	0.92
AE	ADE	6	931.65	364	943.65	1	0.50
DE	ADE	6	931.32	364	943.32	1	0.73
E	ADE	5	999.55	365	1009.55	2	<0.01
**HbA1c**							
18-50							
Saturated model		13	2692.73	1143	2718.73		
ACE	Saturated model	8	2706.91	1148	2722.91	5	0.01
AE	ACE	7	2707.84	1149	2721.84	1	0.33
CE	ACE	7	2730.58	1149	2744.58	1	0.00
E	ACE	6	2961.04	1150	2973.04	2	0.00
50-60							
Saturated model		13	1230.87	465	1256.87		
ACE	Saturated model	8	1235.48	470	1251.48	5	0.47
AE	ACE	7	1236.95	471	1250.95	1	0.22
CE	ACE	7	1235.48	471	1249.48	1	1.00
E	ACE	6	1259.63	472	1271.63	2	0.00
>60							
Saturated model		13	860.92	347	886.92		
ACE	Saturated model	8	864.48	352	880.48	5	0.61
AE	ACE	7	864.48	353	878.48	1	0.99
CE	ACE	7	865.68	353	879.68	1	0.27
E	ACE	6	881.68	354	893.68	2	0.00
**FBG**							
18-50							
Saturated model		13	3297.24	1285	3323.24		

Model	Comparison Model	ep	-2LL	df	AIC	Δdf	P-values
ACE	Saturated model	8	3307.18	1290	3323.18	5	0.08
AE	ACE	7	3308.74	1291	3322.74	1	0.21
CE	ACE	7	3315.90	1291	3329.90	1	0.00
E	ACE	6	3479.23	1292	3491.23	2	0.00
50-60							
Saturated model		13	1301.62	511	1327.62		
ADE	Saturated model	8	1320.04	516	1336.04	5	0.00
AE	ADE	7	1320.04	517	1334.04	1	1.00
DE	ADE	7	1324.42	517	1338.42	1	0.04
E	ACE	6	1365.03	518	1377.03	2	0.00
>60							
Saturated model		13	867.25	341	893.25		
ADE	Saturated model	8	873.14	346	889.14	5	0.32
AE	ADE	7	873.14	347	887.14	1	1.00
DE	ADE	7	876.50	347	890.50	1	0.07
E	ADE	6	898.61	348	910.61	2	0.00
**SBP**							
18-50							
Saturated model		12	3394.79	1350	3418.79		
ACE	Saturated model	7	3400.30	1355	3414.30	5	0.36
AE	ACE	6	3400.67	1356	3412.67	1	0.54
CE	ACE	6	3422.72	1356	3434.72	1	<0.01
E	ACE	5	3654.46	1357	3664.46	2	<0.01
50-60							
Saturated model		12	1539.17	568	1563.17		
ACE	Saturated model	7	1540.56	573	1554.56	5	0.93
AE	ACE	6	1540.78	574	1552.78	1	0.64
CE	ACE	6	1547.03	574	1559.03	1	0.01
E	ACE	5	1630.29	575	1640.29	2	<0.01
>60							
Saturated model		12	1010.51	358	1034.51		
ACE	Saturated model	7	1012.51	363	1026.51	5	0.85
AE	ACE	6	1013.82	364	1025.82	1	0.25
CE	ACE	6	1012.52	364	1024.52	1	0.90
E	ACE	5	1046.19	365	1056.19	2	<0.01
**DBP**							
18-50							

Model	Comparison Model	ep	-2LL	df	AIC	Δdf	P-values
Saturated model		12	3400.08	1348	3424.08		
ACE	Saturated model	7	3408.41	1353	3422.41	5	0.14
AE	ACE	6	3408.79	1354	3420.79	1	0.53
CE	ACE	6	3425.73	1354	3437.73	1	<0.01
E	ACE	5	3629.02	1355	3639.02	2	<0.01
50-60							
Saturated model		12	1583.29	584	1607.29		
ACE	Saturated model	7	1588.34	589	1602.34	5	0.41
AE	ACE	6	1591.71	590	1603.71	1	0.07
CE	ACE	6	1590.04	590	1602.04	1	0.19
E	ACE	5	1687.33	591	1697.33	2	<0.01
>60							
Saturated model		12	1023.39	374	1047.39		
ADE	Saturated model	7	1024.67	379	1038.67	5	0.94
AE	ADE	6	1024.71	380	1036.71	1	0.83
DE	ADE	6	1025.13	380	1037.13	1	0.50
E	ADE	5	1075.20	381	1085.20	2	<0.01
**TC**							
18-50							
Saturated model		13	3389.12	1309	3415.12		
ADE	Saturated model	8	3390.52	1314	3406.52	5	0.92
AE	ADE	7	3390.52	1315	3404.52	1	1.00
DE	ADE	7	3431.41	1315	3445.41	1	0.00
E	ADE	6	3680.79	1316	3692.79	2	0.00
50-60							
Saturated model		13	1500.63	553	1526.63		
ACE	Saturated model	8	1502.28	558	1518.28	5	0.90
AE	ACE	7	1504.51	559	1518.51	1	0.13
CE	ACE	7	1503.91	559	1517.91	1	0.20
E	ACE	6	1589.86	560	1601.86	2	0.00
>60							
Saturated model		13	972.44	359	998.44		
ADE	Saturated model	8	977.09	364	993.09	5	0.46
AE	ADE	7	977.23	365	991.23	1	0.71
DE	ADE	7	979.76	365	993.76	1	0.10
E	ADE	6	1041.36	366	1053.36	2	0.00

Model	Comparison Model	ep	-2LL	df	AIC	Δdf	P-values
**TG**							
18-50							
Saturated model		13	3372.28	1281	3398.28		
ACE	Saturated model	8	3375.70	1286	3391.70	5	0.64
AE	ACE	7	3376.29	1287	3390.29	1	0.44
CE	ACE	7	3396.44	1287	3410.44	1	0.00
E	ACE	6	3604.39	1288	3616.39	2	0.00
50-60							
Saturated model		13	1537.57	549	1563.57		
ADE	Saturated model	8	1545.03	554	1561.03	5	0.19
AE	ADE	7	1545.03	555	1559.03	1	1.00
DE	ADE	7	1554.17	555	1568.17	1	0.00
E	ADE	6	1605.31	556	1617.31	2	0.00
>60							
Saturated model		13	992.06	357	1018.06		
ACE	Saturated model	8	1000.04	362	1016.04	5	0.16
AE	ACE	7	1000.04	363	1014.04	1	0.97
CE	ACE	7	1002.81	363	1016.81	1	0.10
E	ACE	6	1035.88	364	1047.88	2	0.00
**LDL-C**							
18-50							
Saturated model		13	3481.90	1319	3507.90		
ADE	Saturated model	8	3493.72	1324	3509.72	5	0.04
AE	ADE	7	3493.72	1325	3507.72	1	1.00
DE	ADE	7	3539.21	1325	3553.21	1	0.00
E	ADE	6	3762.49	1326	3774.49	2	0.00
50-60							
Saturated model		13	1551.64	555	1577.64		
ACE	Saturated model	8	1554.34	560	1570.34	5	0.75
AE	ACE	7	1557.06	561	1571.06	1	0.10
CE	ACE	7	1554.90	561	1568.90	1	0.45
E	ACE	6	1617.54	562	1629.54	2	0.00
>60							
Saturated model		13	975.64	353	1001.64		
ACE	Saturated model	8	979.04	358	995.04	5	0.64
AE	ACE	7	979.40	359	993.40	1	0.55

Model	Comparison Model	ep	-2LL	df	AIC	Δdf	P-values
CE	ACE	7	981.23	359	995.23	1	0.14
E	ACE	6	1037.88	360	1049.88	2	0.00
**HDL-C**							
18-50							
Saturated model		13	3381.07	1315	3407.07		
ACE	Saturated model	8	3385.49	1320	3401.49	5	0.49
AE	ACE	7	3386.40	1321	3400.40	1	0.34
CE	ACE	7	3425.59	1321	3439.59	1	0.00
E	ACE	6	3738.58	1322	3750.58	2	0.00
50-60							
Saturated model		13	1486.35	557	1512.35		
ACE	Saturated model	8	1487.32	562	1503.32	5	0.97
AE	ACE	7	1489.42	563	1503.42	1	0.15
CE	ACE	7	1491.87	563	1505.87	1	0.03
E	ACE	6	1603.60	564	1615.60	2	0.00
>60							
Saturated model		13	913.91	353	939.91		
ADE	Saturated model	8	917.46	358	933.46	5	0.62
AE	ADE	7	917.46	359	931.46	1	1.00
DE	ADE	7	931.60	359	945.60	1	0.00
E	ADE	6	1010.94	360	1022.94	2	0.00

Abbreviations: BMI, body mass index; HbA1c, glycated hemoglobin; FBG, fasting blood glucose; SBP, systolic blood pressure; DBP, diastolic blood pressure; TC, total cholesterol; TG, plasma triglyceride; LDL-C, low-density lipoprotein-cholesterol; HDL-C, high-density lipoprotein-cholesterol.

*ep, parameters that were free to be estimated; -2LL, negative log-likelihood; df, degrees of freedom; AIC, Akaike's Information Criteria, an alternative fit index. Lower values indicate better model fits; Δdf, the difference in degrees of freedom compared to the saturated model.

*Saturated model, baseline models that estimate the variances and means separately for each twin in a pair and across zygosity; A, additive genetic variance; D, nonadditive genetic variance; C, common environmental variance; E, unique environmental variance.

Supplementary Table S8 Assumptions testing of bivariate SEM for correlations between BMI and cardiometabolic risk factors across age groups

Model	ep	-2LL	df	AIC	Δdf	P-values
**BMI&HbA1c**						
Saturated model	34	9884.09	3830	9952.09		
Submodel 1	30	9884.21	3834	9944.21	4	1.00
Submodel 2	26	9890.59	3838	9942.59	8	0.59
Submodel 3	22	9901.92	3842	9945.92	12	0.12
**BMI&FBG**						
Saturated model	34	10842.59	4194	10910.59		
Submodel 1	30	10845.41	4198	10905.41	4	0.59
Submodel 2	26	10847.49	4202	10899.49	8	0.77
Submodel 3	22	10862.99	4206	10906.99	12	0.06
**BMI&SBP**						
Saturated model	32	11390.55	4460	11454.55		
Submodel 1	28	11393.45	4464	11449.45	4	0.57
Submodel 2	24	11394.17	4468	11442.17	8	0.89
Submodel 3	20	11413.17	4472	11453.17	12	0.03
**BMI&DBP**						
Saturated model	32	11501.68	4516	11565.68		
Submodel 1	28	11507.40	4520	11563.40	4	0.22
Submodel 2	24	11512.68	4524	11560.68	8	0.20
Submodel 3	20	11528.68	4528	11568.68	12	0.01
**BMI&TC**						
Saturated model	34	11203.17	4362	11271.17		
Submodel 1	30	11204.16	4366	11264.16	4	0.91
Submodel 2	26	11206.56	4370	11258.56	8	0.91
Submodel 3	22	11217.50	4374	11261.50	12	0.28
**BMI&TG**						
Saturated model	34	10994.96	4294	11062.96		
Submodel 1	30	10995.54	4298	11055.54	4	0.97
Submodel 2	26	10997.42	4302	11049.42	8	0.96
Submodel 3	22	11004.10	4306	11048.10	12	0.69
**BMI&LDL-C**						
Saturated model	34	11370.92	4374	11438.92		
Submodel 1	30	11371.64	4378	11431.64	4	0.95
Submodel 2	26	11381.74	4382	11433.74	8	0.21
Submodel 3	22	11388.01	4386	11432.01	12	0.15
**BMI&HDL-C**						

Model	ep	-2LL	df	AIC	Δdf	P-values
Saturated model	34	11058.42	4366	11126.42		
Submodel 1	30	11059.01	4370	11119.01	4	0.96
Submodel 2	26	11060.99	4374	11112.99	8	0.96
Submodel 3	22	11067.23	4378	11111.23	12	0.72

Abbreviations: BMI, body mass index; HbA1c, glycated hemoglobin; FBG, fasting blood glucose; SBP, systolic blood pressure; DBP, diastolic blood pressure; TC, total cholesterol; TG, plasma triglyceride; LDL-C, low-density lipoprotein-cholesterol; HDL-C, high-density lipoprotein-cholesterol.

*ep, parameters that were free to be estimated; -2LL, negative log-likelihood; df, degrees of freedom; AIC, Akaike's Information Criteria, an alternative fit index. Lower values indicate better model fits; Δdf, the difference in degrees of freedom compared to the saturated model.

*Saturated model, baseline models that estimate the variances and means separately for each twin in a pair and across zygosity; Submodel 1 equates means within twin pairs; Submodel 2 equates means and variance within twin pairs; Submodel 3 equates means and variances across zygosity.

Supplementary Table S9 Bivariate SEM fits statistics for correlations between BMI and cardiometabolic risk factors across age groups

Model	Comparison Model	ep	-2LL	df	AIC	Δdf	P-values
**BMI&HbA1c**							
ACE	Saturated model	17	9966.93	3847	10000.93	17	0.00
Drop c11	ACE	16	9966.93	3848	9998.93	1	1.00
Drop c22	ACE	16	9977.11	3848	10009.11	1	0.00
Drop c	ACE	15	9977.11	3849	10007.11	2	0.01
Drop c21	ACE	15	9966.93	3849	9996.93	2	1.00
**BMI&FBG**							
ACE	Saturated model	17	10873.11	4211	10907.11	17	0.02
Drop c11	ACE	16	10873.52	4212	10905.52	1	0.52
Drop c22	ACE	16	10877.03	4212	10909.03	1	0.05
Drop c	ACE	15	10877.43	4213	10907.43	2	0.11
Drop c21	ACE	15	10873.52	4213	10903.52	2	0.81
**BMI&SBP**							
ACE	Saturated model	15	11421.63	4477	11451.63	17	0.02
Drop c11	ACE	14	11421.86	4478	11449.86	1	0.63
Drop c22	ACE	14	11438.49	4478	11466.49	1	<0.01
Drop c	ACE	13	11438.77	4479	11464.77	2	<0.01
Drop c21	ACE	13	11421.86	4479	11447.86	2	0.89
**BMI&DBP**							

Model	Comparison Model	ep	-2LL	df	AIC	Δdf	P-values
ACE	Saturated model	15	11534.49	4533	11564.49	17	0.01
Drop c11	ACE	14	11534.65	4534	11562.65	1	0.69
Drop c22	ACE	14	11545.12	4534	11573.12	1	<0.01
Drop c	ACE	13	11545.42	4535	11571.42	2	<0.01
Drop c21	ACE	13	11534.65	4535	11560.65	2	0.92
**BMI&TC**							
ACE	Saturated model	17	11224.69	4379	11258.69	17	0.20
Drop c11	ACE	16	11224.72	4380	11256.72	1	0.86
Drop c22	ACE	16	11227.34	4380	11259.34	1	0.10
Drop c	ACE	15	11227.38	4381	11257.38	2	0.26
Drop c21	ACE	15	11224.72	4381	11254.72	2	0.98
**BMI&TG**							
ACE	Saturated model	17	11008.35	4311	11042.35	17	0.71
Drop c11	ACE	16	11008.35	4312	11040.35	1	0.96
Drop c22	ACE	16	11009.36	4312	11041.36	1	0.31
Drop c	ACE	15	11009.38	4313	11039.38	2	0.60
AE	ACE	15	11008.35	4313	11038.35	2	1.00
**BMI&LDL-C**							
ACE	Saturated model	17	11396.53	4391	11430.53	17	0.08
Drop c11	ACE	16	11396.55	4392	11428.55	1	0.88
Drop c22	ACE	16	11397.07	4392	11429.07	1	0.46
Drop c	ACE	15	11397.09	4393	11427.09	2	0.75
AE	ACE	15	11396.55	4393	11426.55	2	0.99
**BMI&HDL-C**							
ACE	Saturated model	17	11073.07	4383	11107.07	17	0.62
Drop c11	ACE	16	11073.13	4384	11105.13	1	0.81
Drop c22	ACE	16	11077.28	4384	11109.28	1	0.04
Drop c	ACE	15	11077.28	4385	11107.28	2	0.12
Drop c21	ACE	15	11073.13	4385	11103.13	2	0.97

Abbreviations: BMI, body mass index; HbA1c, glycated hemoglobin; FBG, fasting blood glucose; SBP, systolic blood pressure; DBP, diastolic blood pressure; TC, total cholesterol; TG, plasma triglyceride; LDL-C, low-density lipoprotein-cholesterol; HDL-C, high-density lipoprotein-cholesterol.

*ep, parameters that were free to be estimated; -2LL, negative log-likelihood; df, degrees of freedom; AIC, Akaike's Information Criteria, an alternative fit index. Lower values indicate better model fits; Δdf, the difference in degrees of freedom compared to the saturated model.

*Saturated model, baseline models that estimate the variances and means separately for each twin in a pair and across zygosity; A, additive genetic variance; D, nonadditive genetic variance; C, common environmental variance; E,

unique environmental variance.

Supplementary Table S10 Genetic, and environmental correlations between BMI and cardiometabolic traits from the best-fitting bivariate models by sex

Correlations	Male Ra	Re	Female Ra	Re	$P_{homogeneity}$
BMI&HbA1c	0.12(0.00,0.32)	0.13(0.07,0.20)	0.25(0.11,0.38)	0.22(0.10,0.34)	<0.01
BMI&FBG	0.19(0.06,0.37)	0.18(0.11,0.26)	0.10(0.00,0.23)	0.15(0.03,0.26)	<0.01
BMI&SBP	0.44(0.14,0.74)	0.19(0.13,0.24)	0.22(0.10,0.33)	0.17(0.06,0.29)	<0.01
BMI&DBP	0.37(0.22,0.71)	0.24(0.18,0.31)	0.30(0.18,0.41)	0.14(0.02,0.25)	0.01
BMI&TC	0.20(0.10,0.32)	0.13(0.05,0.22)	0.12(0.00,0.25)	0.16(0.05,0.27)	<0.01
BMI&TG	0.45(0.34,0.61)	0.32(0.23,0.41)	0.20(0.05,0.41)	0.27(0.17,0.42)	<0.01
BMI&LDL-C	0.11(0.01,0.20)	0.16(0.07,0.24)	0.17(0.05,0.29)	0.12(0.01,0.23)	<0.01
BMI&HDL-C	-0.35(-0.46,-0.26)	-0.19(-0.28,-0.11)	-0.30(-0.67,-0.11)	-0.09(-0.17,-0.01)	0.02

Abbreviations: Ra, genetic correlations; Re, environmental correlations; BMI, body mass index; HbA1c, glycated hemoglobin; FBG, fasting blood glucose; SBP, systolic blood pressure; DBP, diastolic blood pressure; TC, total cholesterol; TG, plasma triglyceride; LDL-C, low-density lipoprotein-cholesterol; HDL-C, high-density lipoprotein-cholesterol.

Supplementary Table S11 Assumptions testing of bivariate SEM for correlations between BMI and cardiometabolic risk factors by age group

Model	ep	-2LL	df	AIC	Δdf	P-values
**BMI&HbA1c**						
18-50						
Saturated model	34	5591.58	2222	5659.58		
Submodel 1	30	5595.94	2226	5655.94	4	0.36
Submodel 2	26	5603.16	2230	5655.16	8	0.17
Submodel 3	22	5608.16	2234	5652.16	12	0.17
50-60						
Saturated model	34	2463.95	894	2531.95		
Submodel 1	30	2466.88	898	2526.88	4	0.57
Submodel 2	26	2470.42	902	2522.42	8	0.60
Submodel 3	22	2474.68	906	2518.68	12	0.55
>60						
Saturated model	34	1748.40	646	1816.40		
Submodel 1	30	1752.54	650	1812.54	4	0.39
Submodel 2	26	1758.35	654	1810.35	8	0.27
Submodel 3	22	1763.99	658	1807.99	12	0.21
**BMI&FBG**						
18-50						

Model	ep	-2LL	df	AIC	Δdf	P-values
Saturated model	34	6448.92	2498	6516.92		
Submodel 1	30	6452.85	2502	6512.85	4	0.41
Submodel 2	26	6460.50	2506	6512.50	8	0.17
Submodel 3	22	6471.46	2510	6515.46	12	0.03
50-60						
Saturated model	34	2663.66	994	2731.66		
Submodel 1	30	2667.35	998	2727.35	4	0.45
Submodel 2	26	2672.62	1002	2724.62	8	0.35
Submodel 3	22	2680.80	1006	2724.80	12	0.14
>60						
Saturated model	34	1677.72	634	1745.72		
Submodel 1	30	1681.11	638	1741.11	4	0.49
Submodel 2	26	1694.25	642	1746.25	8	0.04
Submodel 3	22	1700.18	646	1744.18	12	0.03
**BMI&SBP**						
18-50						
Saturated model	32	6666.99	2628	6730.99		
Submodel 1	28	6670.53	2632	6726.53	4	0.47
Submodel 2	24	6671.76	2636	6719.76	8	0.78
Submodel 3	20	6681.68	2640	6721.68	12	0.26
50-60						
Saturated model	32	2901.72	1100	2965.72		
Submodel 1	28	2906.03	1104	2962.03	4	0.37
Submodel 2	24	2906.41	1108	2954.41	8	0.79
Submodel 3	20	2910.68	1112	2950.68	12	0.71
>60						
Saturated model	32	1829.28	668	1893.28		
Submodel 1	28	1829.57	672	1885.57	4	0.99
Submodel 2	24	1831.54	676	1879.54	8	0.97
Submodel 3	20	1841.24	680	1881.24	12	0.45
**BMI&DBP**						
18-50						
Saturated model	32	6634.53	2624	6698.53		
Submodel 1	28	6637.57	2628	6693.57	4	0.55
Submodel 2	24	6640.55	2632	6688.55	8	0.65
Submodel 3	20	6651.49	2636	6691.49	12	0.15
50-60						
Saturated model	32	2979.04	1132	3043.04		
Submodel 1	28	2986.53	1136	3042.53	4	0.11
Submodel 2	24	2989.47	1140	3037.47	8	0.24

Model	ep	-2LL	df	AIC	Δdf	P-values
Submodel 3	20	2993.55	1144	3033.55	12	0.27
>60						
Saturated model	32	1869.06	696	1933.06		
Submodel 1	28	1869.99	700	1925.99	4	0.92
Submodel 2	24	1874.59	704	1922.59	8	0.70
Submodel 3	20	1881.43	708	1921.43	12	0.42
**BMI&TC**						
18-50						
Saturated model	34	6517.38	2550	6585.38		
Submodel 1	30	6518.79	2554	6578.79	4	0.84
Submodel 2	26	6520.35	2558	6572.35	8	0.94
Submodel 3	22	6526.10	2562	6570.10	12	0.73
50-60						
Saturated model	34	2866.37	1074	2934.37		
Submodel 1	30	2869.38	1078	2929.38	4	0.56
Submodel 2	26	2870.59	1082	2922.59	8	0.84
Submodel 3	22	2878.67	1086	2922.67	12	0.42
>60						
Saturated model	34	1800.45	670	1868.45		
Submodel 1	30	1801.27	674	1861.27	4	0.94
Submodel 2	26	1803.55	678	1855.55	8	0.93
Submodel 3	22	1810.20	682	1854.20	12	0.64
**BMI&TG**						
18-50						
Saturated model	34	6323.37	2494	6391.37		
Submodel 1	30	6326.02	2498	6386.02	4	0.62
Submodel 2	26	6328.00	2502	6380.00	8	0.80
Submodel 3	22	6330.79	2506	6374.79	12	0.83
50-60						
Saturated model	34	2855.77	1066	2923.77		
Submodel 1	30	2864.27	1070	2924.27	4	0.08
Submodel 2	26	2867.48	1074	2919.48	8	0.16
Submodel 3	22	2876.79	1078	2920.79	12	0.05
>60						
Saturated model	34	1799.56	666	1867.56		
Submodel 1	30	1801.89	670	1861.89	4	0.68
Submodel 2	26	1805.68	674	1857.68	8	0.63
Submodel 3	22	1809.16	678	1853.16	12	0.65
**BMI&LDL-C**						
18-50						

**Model**	**ep**	**-2LL**	**df**	**AIC**	**Δdf**	**P-values**
Saturated model	34	6636.84	2570	6704.84		
Submodel 1	30	6638.55	2574	6698.55	4	0.79
Submodel 2	26	6649.29	2578	6701.29	8	0.13
Submodel 3	22	6652.67	2582	6696.67	12	0.20
50-60						
Saturated model	34	2928.48	1078	2996.48		
Submodel 1	30	2931.46	1082	2991.46	4	0.56
Submodel 2	26	2933.22	1086	2985.22	8	0.78
Submodel 3	22	2936.47	1090	2980.47	12	0.79
>60						
Saturated model	34	1777.53	658	1845.53		
Submodel 1	30	1779.83	662	1839.83	4	0.68
Submodel 2	26	1782.18	666	1834.18	8	0.79
Submodel 3	22	1786.42	670	1830.42	12	0.71
**BMI&HDL-C**						
18-50						
Saturated model	34	6487.54	2558	6555.54		
Submodel 1	30	6489.19	2562	6549.19	4	0.80
Submodel 2	26	6493.19	2566	6545.19	8	0.69
Submodel 3	22	6497.40	2570	6541.40	12	0.63
50-60						
Saturated model	34	2846.00	1082	2914.00		
Submodel 1	30	2850.12	1086	2910.12	4	0.39
Submodel 2	26	2850.84	1090	2902.84	8	0.77
Submodel 3	22	2854.50	1094	2898.50	12	0.74
>60						
Saturated model	34	1719.16	658	1787.16		
Submodel 1	30	1720.84	662	1780.84	4	0.79
Submodel 2	26	1723.58	666	1775.58	8	0.82
Submodel 3	22	1729.53	670	1773.53	12	0.58

Abbreviations: BMI, body mass index; HbA1c, glycated hemoglobin; FBG, fasting blood glucose; SBP, systolic blood pressure; DBP, diastolic blood pressure; TC, total cholesterol; TG, plasma triglyceride; LDL-C, low-density lipoprotein-cholesterol; HDL-C, high-density lipoprotein-cholesterol.

*ep, parameters that were free to be estimated; -2LL, negative log-likelihood; df, degrees of freedom; AIC, Akaike's Information Criteria, an alternative fit index. Lower values indicate better model fits; Δdf, the difference in degrees of freedom compared to the saturated model.

*Saturated model, baseline models that estimate the variances and means separately for each twin in a pair and across zygosity; Submodel 1 equates means within twin pairs; Submodel 2 equates means and variance within twin pairs; Submodel 3 equates means and variances across zygosity.

Supplementary Table S12 Bivariate SEM fits statistics for correlations between BMI and cardiometabolic risk factors by age group

Model	Comparison Model	ep	-2LL	df	AIC	Δdf	P-values
**BMI&HbA1c**							
18-50							
ACE	Saturated model	17	5611.43	2239	5645.43	17	0.28
Drop c11	ACE	16	5611.43	2240	5643.43	1	0.98
Drop c22	ACE	16	5614.17	2240	5646.17	1	0.10
Drop c	ACE	15	5614.17	2241	5644.17	2	0.25
Drop c21	ACE	15	5611.43	2241	5641.43	2	1.00
50-60							
ACE	Saturated model	17	2482.57	911	2516.57	17	0.35
Drop c11	ACE	16	2482.57	912	2514.57	1	1.00
Drop c22	ACE	16	2486.19	912	2518.19	1	0.06
Drop c	ACE	15	2486.19	913	2516.19	2	0.16
AE	ACE	14	2486.19	914	2514.19	3	0.31
Hom	AE	13	2499.52	915	2525.52	2	<0.01
>60							
ACE	Saturated model	17	1769.75	663	1803.75	17	0.21
Drop c11	ACE	16	1770.10	664	1802.10	1	0.55
Drop c22	ACE	16	1771.24	664	1803.24	1	0.22
Drop c	ACE	15	1771.48	665	1801.48	2	0.42
AE	ACE	14	1771.48	666	1799.48	3	0.63
Hom	AE	13	1774.72	667	1800.72	2	0.09
**BMI&FBG**							
18-50							
ACE	Saturated model	17	6473.97	2515	6507.97	17	0.09
Drop c11	ACE	16	6474.49	2516	6506.49	1	0.47
Drop c22	ACE	16	6476.87	2516	6508.87	1	0.09
Drop c	ACE	15	6477.48	2517	6507.48	2	0.17
Drop c21	ACE	15	6474.49	2517	6504.49	2	0.77
50-60							
ACE	Saturated model	17	2690.32	1011	2724.32	17	0.06
Drop c11	ACE	16	2690.32	1012	2722.32	1	1.00
Drop c22	ACE	16	2691.75	1012	2723.75	1	0.23
Drop c	ACE	15	2691.75	1013	2721.75	2	0.49
AE	ACE	14	2691.75	1014	2719.75	3	0.70

Model	Comparison Model	ep	-2LL	df	AIC	Δdf	P-values
Hom	AE	13	2715.12	1015	2741.12	2	<0.01
>60							
ACE	Saturated model	17	1712.04	651	1746.04	17	0.01
Drop c11	ACE	16	1712.16	652	1744.16	1	0.73
Drop c22	ACE	16	1712.04	652	1744.04	1	1.00
Drop c	ACE	15	1712.16	653	1742.16	2	0.94
AE	ACE	14	1712.16	654	1740.16	3	0.99
Hom	AE	13	1725.58	655	1751.58	2	<0.01
**BMI&SBP**							
18-50							
ACE	Saturated model	15	6685.96	2645	6715.96	17	0.33
Drop c11	ACE	14	6686.42	2646	6714.42	1	0.50
Drop c22	ACE	14	6689.66	2646	6717.66	1	0.05
Drop c	ACE	13	6690.34	2647	6716.34	2	0.11
Drop c21	ACE	13	6686.42	2647	6712.42	2	0.79
50-60							
ACE	Saturated model	15	2913.23	1117	2943.23	17	0.83
Drop c11	ACE	14	2913.23	1118	2941.23	1	1.00
Drop c22	ACE	14	2913.97	1118	2941.97	1	0.39
Drop c	ACE	13	2913.97	1119	2939.97	2	0.69
AE	ACE	12	2913.97	1120	2937.97	3	0.87
Hom	AE	11	2948.53	1121	2970.53	1	<0.01
>60							
ACE	Saturated model	15	1844.61	685	1874.61	17	0.57
Drop c11	ACE	14	1844.61	686	1872.61	1	1.00
Drop c22	ACE	14	1847.24	686	1875.24	1	0.11
Drop c	ACE	13	1847.24	687	1873.24	2	0.27
AE	ACE	12	1847.24	688	1871.24	3	0.45
Hom	AE	11	1853.64	689	1875.64	1	0.01
**BMI&DBP**							
18-50							
ACE	Saturated model	15	6657.50	2641	6687.50	17	0.15
Drop c11	ACE	14	6658.03	2642	6686.03	1	0.47
Drop c22	ACE	14	6661.71	2642	6689.71	1	0.04
Drop c	ACE	13	6662.58	2643	6688.58	2	0.08
Drop c21	ACE	13	6658.03	2643	6684.03	2	0.77
50-60							
ACE	Saturated model	15	2995.97	1149	3025.97	17	0.46
Drop c11	ACE	14	2996.15	1150	3024.15	1	0.67
Drop c22	ACE	14	2999.23	1150	3027.23	1	0.07
Drop c	ACE	13	2999.23	1151	3025.23	2	0.20
AE	ACE	12	2999.23	1152	3023.23	3	0.35

Model	Comparison Model	ep	-2LL	df	AIC	Δdf	P-values
Hom	AE	11	3026.40	1153	3048.40	1	<0.01
>60							
ACE	Saturated model	15	1884.49	713	1914.49	17	0.56
Drop c11	ACE	14	1884.49	714	1912.49	1	1.00
Drop c22	ACE	14	1884.83	714	1912.83	1	0.56
Drop c	ACE	13	1884.83	715	1910.83	2	0.84
AE	ACE	12	1884.83	716	1908.83	3	0.95
Hom	AE	11	1896.31	717	1918.31	1	<0.01
**BMI&TC**							
18-50							
ACE	Saturated model	17	6530.78	2567	6564.78	17	0.71
Drop c11	ACE	16	6530.88	2568	6562.88	1	0.76
Drop c22	ACE	16	6531.01	2568	6563.01	1	0.63
Drop c	ACE	15	6531.11	2569	6561.11	2	0.85
AE	ACE	14	6531.11	2570	6559.11	3	0.96
50-60							
ACE	Saturated model	17	2881.95	1091	2915.95	17	0.55
Drop c11	ACE	16	2881.95	1092	2913.95	1	1.00
Drop c22	ACE	16	2885.57	1092	2917.57	1	0.06
Drop c	ACE	15	2885.57	1093	2915.57	2	0.16
AE	ACE	14	2885.57	1094	2913.57	3	0.31
Hom	AE	13	2909.71	1095	2935.71	2	<0.01
>60							
ACE	Saturated model	17	1823.86	687	1857.86	17	0.14
Drop c11	ACE	16	1823.86	688	1855.86	1	1.00
Drop c22	ACE	16	1823.98	688	1855.98	1	0.73
Drop c	ACE	15	1823.98	689	1853.98	2	0.94
AE	ACE	14	1823.98	690	1851.98	3	0.99
Hom	AE	13	1837.01	691	1863.01	2	<0.01
**BMI&TG**							
18-50							
ACE	Saturated model	17	6334.03	2511	6368.03	17	0.87
Drop c11	ACE	16	6334.17	2512	6366.17	1	0.71
Drop c22	ACE	16	6336.35	2512	6368.35	1	0.13
Drop c	ACE	15	6336.65	2513	6366.65	2	0.27
Drop c21	ACE	15	6334.17	2513	6364.17	2	0.93
50-60							
ACE	Saturated model	17	2880.77	1083	2914.77	17	0.09
Drop c11	ACE	16	2880.77	1084	2912.77	1	1.00
Drop c22	ACE	16	2880.77	1084	2912.77	1	1.00
Drop c	ACE	15	2880.77	1085	2910.77	2	1.00
AE	ACE	14	2880.77	1086	2908.77	3	1.00

Model	Comparison Model	ep	-2LL	df	AIC	Δdf	P-values
Hom	AE	13	2915.16	1087	2941.16	2	<0.01
>60							
ACE	Saturated model	17	1812.62	683	1846.62	17	0.73
Drop c11	ACE	16	1812.62	684	1844.62	1	1.00
Drop c22	ACE	16	1812.80	684	1844.80	1	0.68
Drop c	ACE	15	1812.80	685	1842.80	2	0.92
AE	ACE	14	1812.80	686	1840.80	3	0.98
Hom	AE	13	1824.63	687	1850.63	2	<0.01
**BMI&LDL-C**							
18-50							
ACE	Saturated model	17	6660.79	2587	6694.79	17	0.12
Drop c11	ACE	16	6660.84	2588	6692.84	1	0.83
Drop c22	ACE	16	6660.79	2588	6692.79	1	1.00
Drop c	ACE	15	6660.84	2589	6690.84	2	0.98
AE	ACE	14	6660.84	2590	6688.84	3	1.00
50-60							
ACE	Saturated model	17	2940.81	1095	2974.81	17	0.78
Drop c11	ACE	16	2940.81	1096	2972.81	1	1.00
Drop c22	ACE	16	2944.95	1096	2976.95	1	0.04
Drop c	ACE	15	2944.95	1097	2974.95	2	0.13
AE	ACE	14	2944.95	1098	2972.95	3	0.25
Hom	AE	13	2963.55	1099	2989.55	2	<0.01
>60							
ACE	Saturated model	17	1800.41	675	1834.41	17	0.15
Drop c11	ACE	16	1800.41	676	1832.41	1	1.00
Drop c22	ACE	16	1800.96	676	1832.96	1	0.46
Drop c	ACE	15	1800.96	677	1830.96	2	0.76
AE	ACE	14	1800.96	678	1828.96	3	0.91
Hom	AE	13	1812.21	679	1838.21	2	<0.01
**BMI&HDL-C**							
18-50							
ACE	Saturated model	17	6503.67	2575	6537.67	17	0.51
Drop c11	ACE	16	6504.11	2576	6536.11	1	0.51
Drop c22	ACE	16	6506.03	2576	6538.03	1	0.12
Drop c	ACE	15	6506.03	2577	6536.03	2	0.31
AE	ACE	14	6506.03	2578	6534.03	3	0.50
50-60							
ACE	Saturated model	17	2865.45	1099	2899.45	17	0.30
Drop c11	ACE	16	2865.45	1100	2897.45	1	1.00
Drop c22	ACE	16	2868.49	1100	2900.49	1	0.08
Drop c	ACE	15	2868.49	1101	2898.49	2	0.22
AE	ACE	14	2868.49	1102	2896.49	3	0.38

Model	Comparison Model	ep	-2LL	df	AIC	Δdf	P-values
Hom	AE	13	2895.10	1103	2921.10	2	<0.01
>60							
ACE	Saturated model	17	1733.36	675	1767.36	17	0.65
Drop c11	ACE	16	1733.36	676	1765.36	1	1.00
Drop c22	ACE	16	1733.36	676	1765.36	1	1.00
Drop c	ACE	15	1733.36	677	1763.36	2	1.00
AE	ACE	14	1733.36	678	1761.36	3	1.00
Hom	AE	13	1754.58	679	1780.58	2	<0.01

Abbreviations: BMI, body mass index; HbA1c, glycated hemoglobin; FBG, fasting blood glucose; SBP, systolic blood pressure; DBP, diastolic blood pressure; TC, total cholesterol; TG, plasma triglyceride; LDL-C, low-density lipoprotein-cholesterol; HDL-C, high-density lipoprotein-cholesterol.

*ep, parameters that were free to be estimated; -2LL, negative log-likelihood; df, degrees of freedom; AIC, Akaike's Information Criteria, an alternative fit index. Lower values indicate better model fits; Δdf, the difference in degrees of freedom compared to the saturated model.

*Saturated model, baseline models that estimate the variances and means separately for each twin in a pair and across zygosity; A, additive genetic variance; D, nonadditive genetic variance; C, common environmental variance; E, unique environmental variance; Hom, homogeneity model, which equates the variance components across age groups.

Supplementary Table S13 Phenotypic, Genetic, and unique environmental correlations between BMI and cardiometabolic risk factors by age group

	Rph	Ra	Re
**BMI&HbA1c**			
18-50	0.23(0.16,0.29)	0.29(0.18,0.42)	0.12(0.05,0.22)
50-60	0.07(0.01,0.17)	0.01(0.00,0.20)	0.15(0.00,0.28)
>60	0.22(0.11,0.33)	0.14(0.00,0.34)	0.33(0.17,0.47)
**BMI&FBG**			
18-50	0.23(0.17,0.29)	0.33(0.20,0.55)	0.13(0.06,0.22)
50-60	0.09(0.01,0.19)	0.08(0.00,0.23)	0.12(0.00,0.26)
>60	0.23(0.13,0.34)	0.09(0.00,0.29)	0.42(0.27,0.55)
**BMI&SBP**			
18-50	0.32(0.26,0.37)	0.44(0.32,0.60)	0.17(0.10,0.26)
50-60	0.24(0.15,0.32)	0.23(0.08,0.37)	0.26(0.13,0.38)
>60	0.16(0.07,0.27)	0.08(0.00, 0.30)	0.26(0.11,0.40)
**BMI&DBP**			
18-50	0.36(0.30,0.41)	0.49(0.36,0.68)	0.22(0.15,0.31)
50-60	0.28(0.19,0.36)	0.31(0.16,0.44)	0.23(0.10,0.35)
>60	0.10(0.04,0.20)	0.00(0.00,0.17)	0.25(0.10,0.38)
**BMI&TC**			
18-50	0.19(0.13,0.25)	0.20(0.11,0.29)	0.18(0.09,0.26)

	Rph	Ra	Re
50-60	0.14(0.05,0.23)	0.09(0.00,0.24)	0.22(0.09,0.35)
>60	0.04(0.00,0.16)	0.07(0.00,0.25)	0.00(0.00,0.16)
**BMI&TG**			
18-50	0.35(0.29,0.41)	0.39(0.27,0.54)	0.33(0.25,0.45)
50-60	0.29(0.20,0.37)	0.32(0.16,0.47)	0.26(0.12,0.38)
>60	0.29(0.18,0.39)	0.30(0.09,0.49)	0.28(0.12,0.42)
**BMI&LDL-C**			
18-50	0.15(0.09,0.21)	0.16(0.06,0.25)	0.15(0.06,0.24)
50-60	0.11(0.04,0.20)	0.04(0.00,0.21)	0.20(0.07,0.33)
>60	0.11(0.00,0.22)	0.14(0.00,0.34)	0.05(0.00,0.22)
**BMI&HDL-C**			
18-50	-0.28(-0.34,-0.22)	-0.30(-0.38,-0.22)	-0.21(-0.30,-0.13)
50-60	-0.21(-0.30,-0.11)	-0.25(-0.39,-0.11)	-0.11(-0.24, 0.00)
>60	-0.22(-0.34,-0.11)	-0.21(-0.39,-0.02)	-0.25(-0.40,-0.09)

Abbreviations: Rph, phenotypic correlations; Ra, genetic correlations; Re, environmental correlations; BMI, body mass index; HbA1c, glycated hemoglobin; FBG, fasting blood glucose; SBP, systolic blood pressure; DBP, diastolic blood pressure; TC, total cholesterol; TG, plasma triglyceride; LDL-C, low-density lipoprotein-cholesterol; HDL-C, high-density lipoprotein-cholesterol.
